# Inhibition of Ctsk modulates periodontitis with arthritis via downregulation of TLR9 and autophagy

**DOI:** 10.1111/cpr.12722

**Published:** 2019-11-18

**Authors:** Wei Wei, Jie Ren, Wuwei Yin, Handong Ding, Qiuyu Lu, Liangyu Tan, Shibing Deng, Jie Liu, Qin Yang, Jiajia Wang, Min Wang, Yuan Yue, Liang Hao

**Affiliations:** ^1^ The State Key Laboratory of Oral Diseases & National Clinical Research Center for Oral Diseases Department of Prosthodontics West China Hospital of Stomatology Sichuan University Sichuan China; ^2^ Stomatological Hospital Southern Medical University Guangzhou China

**Keywords:** arthritis, autophagy, cathepsin K, periodontitis, Toll‐like receptor 9

## Abstract

**Objectives:**

The mechanisms underlying the effects of Toll‐like receptor 9 (TLR9) and autophagy on rheumatoid arthritis (RA)‐aggravated periodontitis are unclear. We aimed to explore a novel target, cathepsin K (Ctsk)‐mediated TLR9‐related autophagy, during the progress of periodontitis with RA.

**Materials and Methods:**

DBA/J1 mouse model of periodontitis with RA was created by local colonization of *Porphyromonas gingivalis *(*Pg*) and injection of collagen. The expression of Ctsk was inhibited by adeno‐associated virus (AAV). Micro‐CT, immunohistochemistry (IHC), Western blot and quantitative real‐time polymerase chain reaction (qRT‐PCR) were used to detect the expression of TLR9‐related autophagy in periodontitis with RA. Small interfering RNA (siRNA) and CpG oligodeoxynucleotides (CpG ODN) were applied in macrophages. Western blot, immunofluorescence (IF) and qRT‐PCR were used to verify the in vivo results.

**Results:**

RA can promote periodontitis bone destruction in the lesion area, while inhibiting Ctsk could effectively alleviate this effect. The infiltration of macrophages, TLR9, autophagy proteins (TFEB and LC3) and inflammatory cytokines increased in the periodontitis‐with‐RA group and was reduced by the inhibition of Ctsk in the periodontal region. Macrophage stimulation confirmed the in vivo results. With the activation of TLR9 by CpG ODN, inhibition of Ctsk could suppress both TLR9 downstream signalling proteins and autophagy‐related proteins.

**Conclusions:**

This study advanced a novel role for Ctsk in TLR9 and autophagy to explain the interaction between periodontitis and RA.

## 1 INTRODUCTION

Periodontitis is a chronic inflammatory disease which has a destructive effect on periodontal supporting tissues.^1^ The main clinical manifestations of periodontitis are attachment loss and alveolar bone resorption. As a chronic inflammatory disease, periodontitis is inseparable from the body's innate and acquired immune systems.^2^ After resisting microbial attack, the host's immune response is suppressed by the pathogen, but the bacteria also activate the immune system by interacting with immune cells and the complement cascade, leading to a persistent inflammatory state.^3^ Current research shows that periodontitis is closely related to autoimmune diseases.^4^

Rheumatoid arthritis (RA), one of the most common autoimmune diseases, is characterized by chronic joint inflammation and bone destruction.^5^ In addition to the specific autoimmune response to citrullinated protein in the body,^6^ the autoimmune pathogenic mechanism of RA involves infiltration of various immune cells in the synovial tissue of diseased joints, the activation of the complement system and the maladjustment of cytokine networks.^7^, ^8^

Periodontitis and RA are both chronic inflammatory diseases with similar immune and inflammatory mechanisms, causing soft tissue inflammation and bone destruction.^9^ A recent study has shown an epidemiological association between periodontitis and RA.^10^ This supported previous evidence showing that chronic periodontitis is more common in patients with RA than in healthy controls^11^ and that the prevalence of RA in patients with chronic periodontitis is higher than in the general population.^12^

For cell physiology and health, autophagy is a necessary conservative lysosomal degradation process.^13^ Autophagy can regulate cellular processes such as apoptosis, inflammation, pathogen clearance and immune response, and is a potential therapeutic target for many diseases.^14^ Through mediating the survival of *Pg*, the autophagy pathway exerts effects on periodontal diseases.^15^ It has also been shown that suppression of the autophagy pathway is related to the pathogenesis of rheumatoid arthritis and other autoimmune diseases.^16^ More specifically, autophagy seems to be involved in the production of citrulline peptide and the apoptosis resistance of RA cells.^17^ Therefore, the substantial progress of autophagy research provides a new understanding of the pathogenesis of periodontitis and RA.

Cathepsin K (Ctsk) is a lysosome cysteine protease that is primarily expressed in osteoclasts and plays a key role in bone resorption.^18^ Ctsk inhibitors have been considered as potential therapies for diseases characterized by relative bone destruction. Our previous studies have shown that inhibition of Ctsk can effectively reduce bone resorption in periodontitis associated with arthritis, and have also found a link between Ctsk and Toll‐like receptor 9 (TLR9). Inhibition of Ctsk has been shown to downregulate the TLR9 pathway.^19^ During the process of bacterial‐induced inflammation, TLR9 can recognize the CpG oligodeoxynucleotide (CpG ODN) presented in bacterial DNA for innate immunity, which is a key pattern recognition receptor for the immune response.^20^ Meanwhile, studies have shown that the TLR9 receptor is involved in autophagy.^21^, ^22^ For example, after TLR9 stimulation, the autophagy protein microtubule‐associated protein 1A/1B‐light chain 3 (LC3) can directly recruit IκB kinase α (IKKα) for type I Interferon (IFN) production.^21^ There is also evidence suggesting that CpG ODN can induce autophagy in rodent and human tumour cell lines via the Toll‐like receptor 9‐extracellular regulated protein kinase‐mammalian target of rapamycin (TLR9‐ERK‐mTOR) signalling pathway.^22^ These data led us to the hypothesis that inhibition of Ctsk could modulate autophagy via downregulation of TLR9 and, in turn, affect the progression of RA‐aggravated periodontitis.

Here, we investigated the relationship between Ctsk and TLR9‐related autophagy in the presence of periodontitis and RA. Our findings provide a new approach and therapeutic target for investigating RA‐aggravated periodontitis.

## 2 MATERIALS AND METHODS

### 2.1 Animals

A total of 80 six‐week‐old male DBA/J1 mice (purchased from the Nanjing Biomedical Research Institute) were used for this study. Mice were randomly allocated into eight groups: (a) Control: normal mice, N = 10; (b) Control + AAV‐GFP: mice received adeno‐associated virus‐GFP‐blank control virus (AAV‐GFP) transfection, N = 10; (c) *Pg* + AAV‐GFP: mice received AAV‐GFP transfection and oral infection with *Porphyromonas gingivalis* (*Pg*), N = 10; (d) *Pg* + AAV‐sh‐Ctsk: mice received adeno‐associated virus expressing cathepsin K small hairpin RNA (AAV‐sh‐Ctsk) transfection and oral infection with *Pg*, N = 10; (e) CIA + AAV‐GFP: mice with collagen‐induced arthritis (CIA) received AAV‐GFP transfection, N = 10; (f) CIA + AAV‐sh‐Ctsk: mice with CIA received AAV‐sh‐Ctsk transfection, N = 10; (g) CIA + *Pg* + AAV‐GFP: mice with CIA received AAV‐GFP transfection and oral infection with *Pg*, N = 10; and (h) CIA + *Pg* + AAV‐sh‐Ctsk: mice with CIA received AAV‐sh‐Ctsk transfection and oral infection with *Pg*, N = 10. This study was approved by the Sichuan University Institutional Animal Care and Use Committee (Protocol number: SKLODLL2014A026). All animals were maintained at the State Key Laboratory for Oral Disease animal facilities and were given standard laboratory mouse food and water.

### 2.2 Experimental periodontitis and collagen‐induced arthritis (CIA)

We established our mouse model of periodontitis with CIA following previous protocols.^19^ The bacterial strain used was *Pg* (ATCC: 33277), which was grown in an anaerobic chamber (80% N_2_, 10% H_2_ and 10% CO_2_) at 37°C. Before the formal oral bacterial colonization, kanamycin (0.5 mg/mL) was added to drinking water for three consecutive days in order to remove other bacteria. The bacterial pellet obtained by centrifugation was mixed with an equal volume of sterile 3% carboxymethylcellulose (CMC) and topically applied in the oral cavity and anus eight consecutive times. The dose of the mixture was 100 μL (5 × 10^10^ cells/mL of *Pg*) per mouse.^23^ Following bacterial application, chicken type II collagen (Cat#20011, Chondrex) was emulsified in 100 μg complete Freund's adjuvant (Cat#7023, Chondrex). Fifty microlitres of the emulsion was slowly injected intradermally at a point about 1.5 cm distal from the base of the tail. The primary immunization was performed one day after bacterial application, and the booster immunization was made after 14 days.^24^

### 2.3 AAV transfection

The AAVs were designed and synthesized by GeneChem. The nucleotide sequence of AAV‐U6‐Ctsk‐shRNA‐CAG‐EGFP was 5′‐ACCGGGAGGTGTGTACTATGATGAAATTCAAGAGATTTCATCATAGTACACACCTCTTTTT‐3′). Empty AAV vectors (AAV‐U6‐CAG‐EGFP) were used as negative control for non‐sequence‐specific effects.^25^ AAV titres were diluted to a working concentration of 2.5 × 10^10^ genomic particles per mL with pre‐cooled sterile PBS. Ten microlitres of AAV working solution was injected into each periodontal region and knee joint using a 100‐μL microsyringe. Mice were transfected with AAV once a day and rested for four days after two consecutive days. The procedure of AAV transfections was repeated eight times during the experimental period.^26^

### 2.4 Cell culture and siRNA transfection reagents

Murine macrophage cell line RAW264.7 was cultured in DMEM high‐glucose medium. According to the instructions (GenePharma), siRNA transfection can be started after 24‐hour cell seeding at a density of 5 × 10^5^ cells per T25 culture flask. The concentration of siRNA was 100 nmol/L per T25 culture flask. Per 1 mL, transfection working solution included 10 μL EndoFectin™ Max transfection reagent and 200 pmol siRNA. After cell seeding in the T25 culture flask, 2 mL transfection working solution was first added into the T25 culture flask, then 2 mL DMEM high‐sugar medium was added 6 hours later to continue the culture. Cells were incubated for 24 hours until the medium was changed for DMEM high glucose with 10% FBS, containing 5 μmol/L CpG ODN or not.^27^ Twenty‐four hours later, cells were collected for RNA and protein extractions.

We used two sets of siRNAs: CtsK‐Mus‐418 siRNA (sense: 5′‐CCUCUCGAUCCUACAGUAATT‐3′; anti‐sense: 5′‐UUACUGUAGGAUCGAGAGGTT‐3′) and negative control siRNA (sense: 5′‐UUCUUCGAACGUGUCACGUTT‐3′; anti‐sense: 5′‐ACGUGACACGUUCGGAGAATT‐3′).

### 2.5 Harvest and preparation of samples

Animals were euthanized by anaesthetic overdose 56 days after initial bacterial infection. Bilateral maxillary and mandibular bones and bilateral knee joints were defleshed and collected. For micro‐CT and histological analysis, right maxillary bone and right knee joints were fixed in 4% paraformaldehyde overnight and washed under running water continuously for 8 hours, and then transferred to 70% alcohol for storage at 4°C. The left maxillary bone and left knee joints for RNA and protein extraction were stored at −80°C.

### 2.6 Micro‐CT scanning

Micro‐CT scanning of maxillae and knee samples was applied by vivaCT 80 (Scanco Medical) with the resolution of 12 μm.^28^ Three‐dimensional reconstruction and data analysis were performed by Scanco Evaluation and Mimics Research 19.0 software. The alveolar bone and subchondral bone area of tibia and femur were defined as the region of interest (ROI), and bone volume/total volume was calculated for quantification.^29^ The region from the cemento‐enamel junction (CEJ) of the molar to the alveolar bone crest (ABC) was defined as the vertical alveolar bone resorption.

### 2.7 Immunohistochemistry (IHC) and immunofluorescence (IF) analysis

The animal specimens for IHC or IF analysis were demineralized and embedded in paraffin wax, and then sectioned into 5 μm thicknesses. Sections were submitted to analysis with F4/80 (Cat#70076, Cell Signaling Technology), TFEB (Cat#A303‐673A, Bethyl Laboratories) and GFP (Cat#2956, Cell Signaling Technology) rabbit monoclonal primary antibody according to the manufacturer's instructions. The sections of IF were then stained with DAPI (Cat#4083, Cell Signaling Technology) to identify the nuclei.^30^ All samples in vitro were fixed by 4% paraformaldehyde solution and then treated with 0.5% Triton X‐100 for 15 minutes and 5% serum for 1 hour. The cells were incubated with TLR9 and TFEB primary antibodies at 4°C overnight. On the second day, cells were incubated with the secondary antibody and stained using phalloidin and DAPI successively. Finally, the samples were sealed with the anti‐fluorescence quenching agent, and the images were observed and captured under the fluorescence microscope.

### 2.8 RNA extraction and qRT‐PCR analysis

Total RNA was extracted from tissue homogenate and cultured cells using NucleoTrap® mRNA Midi (Cat#740656, TaKaRa) according to the manufacturer's instructions. Reversed transcription of total RNA was carried out using RevertAid First Strand cDNA Synthesis Kit (Cat#K1622, Thermo Fisher). Following the standardized protocol from the manufacturer, 96‐well Optical Reaction Plate (Cat. #4309849, ABI) and SYBR® Green PCR MasterMix (Cat. #S4438, Sigma) were used for qRT‐PCR. The primers used to detect the expression level of different genes are shown in Table 1.

**Table 1:** Primers used in qRT‐PCR

Gene	Forward primers (from 5′ to 3′)	Reverse primers (from 5′ to 3′)
*Tnfα*	AGGGTCTGGGCCATAGAACT	CCACCACGCTCTTCTGTCTAC
*Il6*	CTCTGCAAGAGACTTCCATCCAGT	GAAGTAGGGAAGGCCGTGG
*Tfeb*	CCACCCCAGCCATCAACAC	CAGACAGATACTCCCGAACCTT
*Ctsk*	CCAGGAAATGAGCTTGACAAA	ATAATTCTCAGTCACACAGTCCACA
*Lc3*	GACCGCTGTAAGGAGGTGC	CTTGACCAACTCGCTCATGTTA
*P62*	AGGATGGGGACTTGGTTGC	TCACAGATCACATTGGGGTGC
*Tlr9*	TTCTCAAGACGGTGGATCGC	TGGAGACAGGCTGAGTGCAA
*Myd88*	AGGACAAACGCCGGAACTTTT	GCCGATAGTCTGTCTGTTCTAGT
*Traf6*	TGTTCTTAGCTGCTGGGGTGT	GAAGGAGCTGGAGAGGTTCC
*Irak1*	CGCCCAGCACTTCTTGTGCGA	GATCAAGGCCGCGAACT
*Irak4*	GTCATGACCAGCCGAATCGTG	CAGACACTGGCTAGCAGCAGA
*Gapdh*	AGGTTGTCTCCTGCGACTTCA	CCAGGAAATGAGCTTGACAAA

### 2.9 Protein extraction and immunoblotting

Total protein of the samples and cells was extracted using Total Protein Extraction Kit (Cat#PE001, SAB), and the concentration of protein in the supernatant was measured using BCA Protein Assay Kit (Cat#P0012S, Beyotime). The extraction concentration of total protein in vivo was 5‐7 mg/mL and 10‐15 mg/mL in vitro*.* After confirming the protein loading amount of each sample was 100 µg, we dissolved the protein samples by SDS‐PAGE and then transferred onto the membrane. The conditions of SDS‐PAGE were constant voltage (60 V) for 2 hours, and the condition of membrane transfer is constant current (200 mA) for 100 minutes. The membrane was blotted with specific primary antibodies, TLR9, TFEB, CTSK, LC3A/B and GAPDH (Cat#13674, Cat#32361, Cat#4980, Cat#12741, Cat#5174, respectively, Cell Signaling Technology). According to the manufacturer's instructions, the concentration of primary antibodies was 1:1000. On the second day, HRP‐conjugated antibodies (Cat#L3012‐2, SAB) were applied to the membranes and the signals were detected using the ChemiDoc™ MP Imaging System (Bio‐Rad).^31^

### 2.10 Safranin O staining (SO)

According to the manufacturer's instructions (Cat#TMS‐009, Sigma Aldrich), after dewaxing and dehydrating the sample sections, the specific process of safranin O staining was performed as follows: slides were stained with 0.002% fast green solution and washed with 1% acetic acid. After stained with haematoxylin solution for 10 minutes and rinsed, the slides were dyed with 0.1% SO solution for 6 minutes. Articular cartilage was evaluated by OARSI grading.^32^

### 2.11 Statistical analysis

Data were shown as mean ± SD of different groups and analysed using two‐tailed Student's *t* test and one‐way ANOVA test followed by Tukey's multiple comparison test and non‐parametric Mann‐Whitney *U* test/Kruskal‐Wallis test. *P* values < .05 or *U* values > 1.96 were considered significant.

## 3 RESULTS

### 3.1 Inhibition of Ctsk in the lesion area reduced bone destruction from periodontitis and comorbid rheumatoid arthritis

**Figure 1:**
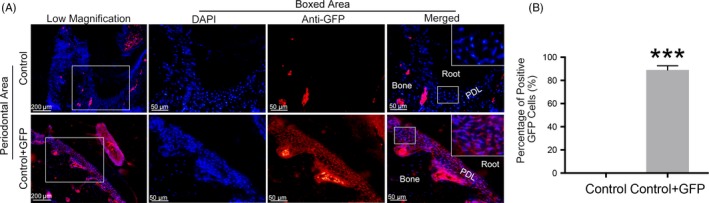
Immunofluorescence analysis of alveolar bone in the control group and the empty AAV vectors (GFP) group. A, IF staining of GFP‐positive cells in periodontal area. Representative images are shown. B, The percentage of GFP‐positive cells in different groups. Anti‐mouse GFP antibody was applied to detect the AAV transfection efficiency. Red spots are GFP‐positive cells. PDL, periodontal ligament. Data are presented as the mean ± SD (n = 10 per group), compared with the control. *P < .05; **P < .01; and ***P < .001

Since the AAVs designed in our study contained the sequence of GFP, we could detect the transfection effect of AAVs by GFP fluorescence staining. Compared with the control group, the percentage of positive GFP cells was increased significantly in the AAV‐treated group (the Control + GFP group) (Figure 1A,B), which indicated that the transfection of AAVs in vivo was successful.

**Figure 2:**
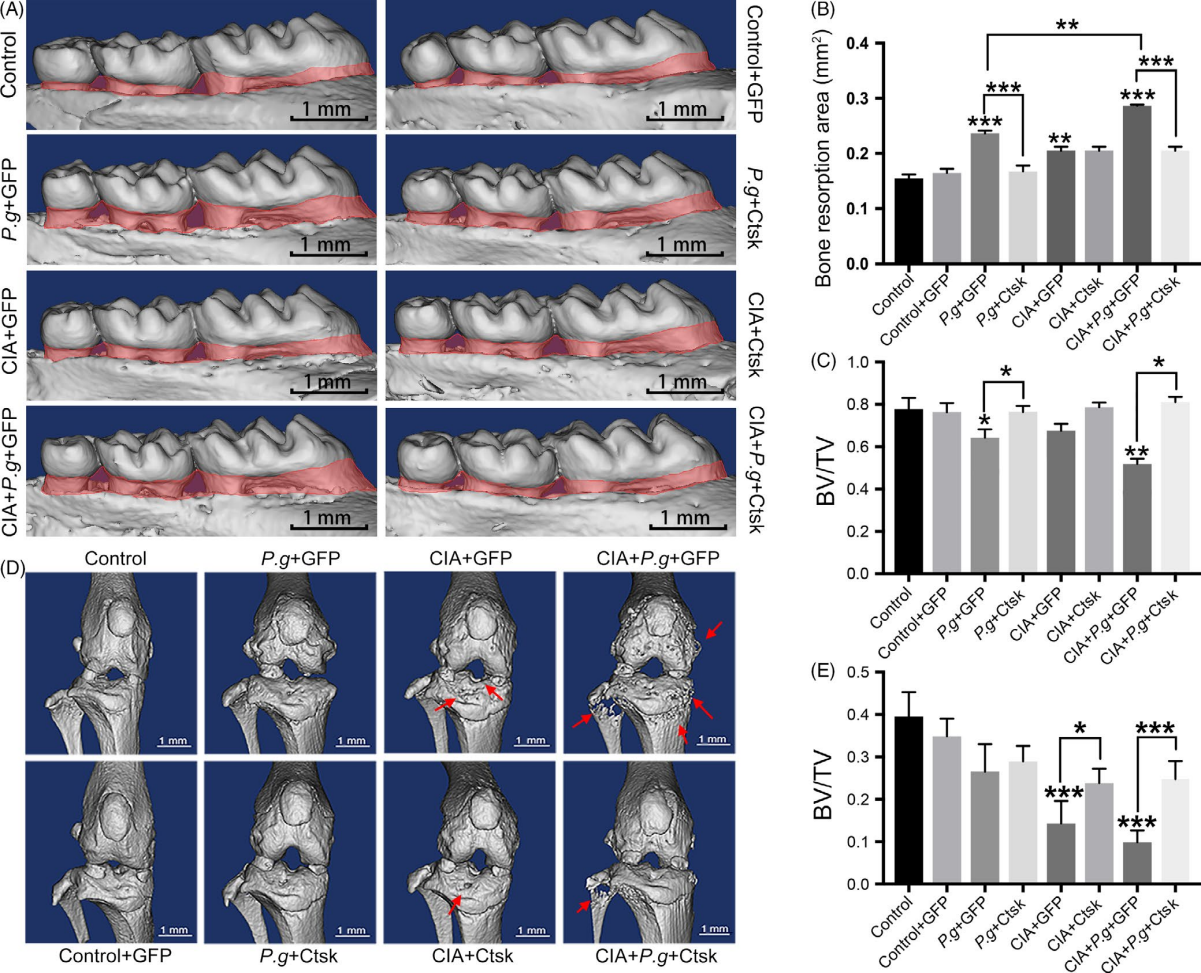
Inhibition of Ctsk in the lesion area reduced bone destruction in periodontitis and comorbid rheumatoid arthritis. A, Three‐dimensional reconstruction of the maxilla samples from different groups. Vertical bone resorption is indicated by the red‐marked area. B, Quantification of vertical bone resorption in different groups. C, Quantification of bone volume/total volume measured for alveolar bone areas in different groups. D, Three‐dimensional reconstruction of knee joints. The red arrow indicates the bone destruction of knee joints. E, Bone volume/total volume of the subchondral bone area of tibia and femur. Data are presented as the mean ± SD (n = 10 per group), compared with the control. Control: untreated DBA/J1 mice; *Pg*: the mice orally infected by *Porphyromonas gingivalis*; CIA: mice with collagen‐induced arthritis (CIA); GFP: mice received empty AAV vectors (AAV‐U6‐CAG‐EGFP); and Ctsk: mice received AAV‐U6‐Ctsk‐shRNA‐CAG‐EGFP. *P < .05, **P < .01, ***P < .001. The asterisk (with no line connection) on the column of this group represents the statistical difference between it and the control group. Experiments were repeated three times

To investigate the effect of Ctsk on bone destruction from periodontitis and comorbid RA, we analysed the alveolar bone and joint in vivo by micro‐CT scanning for bone malformation in lesion areas. According to the images of alveolar bones scanned by micro‐CT, we quantitated and analysed periodontal bone resorption. The results showed a greater bone resorption area in the periodontitis group (*Pg* + GFP) than in the control group (Figure 2A,B), which indicated that the model of periodontitis had been successfully established. The bone resorption area in the group with periodontitis and CIA (CIA + *Pg* + GFP) was larger than that in the periodontitis group (*Pg* + GFP). Consistent with our hypothesis, oral transfection with AAV‐sh‐Ctsk reversed alveolar bone resorption in the periodontitis and comorbid groups, and there was no significant difference among groups with inhibition of Ctsk. The trends in bone volume/total volume (BV/TV) were similar to those of bone resorption (Figure 2C).

**Figure 3:**
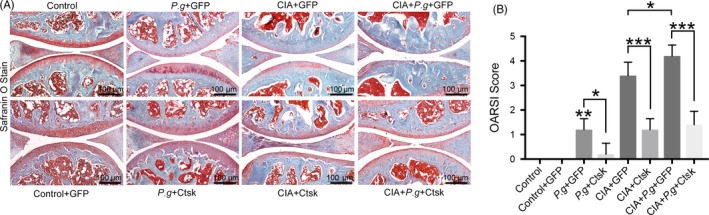
Safranin O staining of knee joints and OARSI score of knee joints with SO staining results. A, Safranin O staining of knee joints in different groups. Representative images are shown. B, OARSI score of knee joints with SO staining in different groups. Data are presented as the mean ± SD (n = 10 per group), compared with the control. Control: untreated DBA/J1 mice; *Pg*: DBA/J1 mice orally infected with *Porphyromonas gingivalis*; CIA: mice with collagen‐induced arthritis (CIA); GFP: mice received empty AAV vectors (AAV‐U6‐CAG‐EGFP); and Ctsk: mice received AAV‐U6‐Ctsk‐shRNA‐CAG‐EGFP. *P < .05, **P < .01, ***P < .001. The asterisk (with no line connection) on the column of this group represents the statistical difference between it and the control group. Experiments were repeated three times

Three‐dimensional reconstruction of knee joints showed varying degrees of bone destruction in the knee area (Figure 2D). The knee joints of mice with periodontitis (*Pg* + GFP) had roughened bone surface compared with the control group. The knee joints of mice with CIA (CIA + GFP) had osteophytes and bone resorption pits on the surface. Mice in the CIA with the periodontitis group (CIA + *Pg* + GFP) suffered the most severe knee joint damage, with the subchondral area of tibia and fibula exhibiting a mesh‐like appearance reflecting bone destruction. Similar to the changes in the periodontal region, intra‐articular AAV‐sh‐Ctsk transfection led to a remission of bone damage in the CIA and comorbidity groups (Figure 2D). BV/TV results were consistent with these findings (Figure 2E). In addition, safranin O staining and OARSI score were used to observe cartilage destruction in knee joints. Similarly, the comorbidity group had the most severe cartilage damage and the highest OARSI score. Inhibiting Ctsk could reduce the destruction of articular cartilage and OARSI score (Figure 3A,B). Taken together, these data demonstrated that periodontitis and arthritis promote one another, while inhibiting Ctsk effectively alleviates bone destruction and the mutual promotion of the two diseases.

### 3.2 Inhibition of Ctsk in the lesion area reduced the number of macrophages and inflammatory cytokines in the periodontium with RA

In order to further explore the influence of periodontitis with RA on the inflammatory state in the lesion area and to explore whether inhibiting Ctsk alleviated the inflammatory state, we evaluated the abundance of macrophages and inflammatory cytokines in the lesion area.

**Figure 4:**
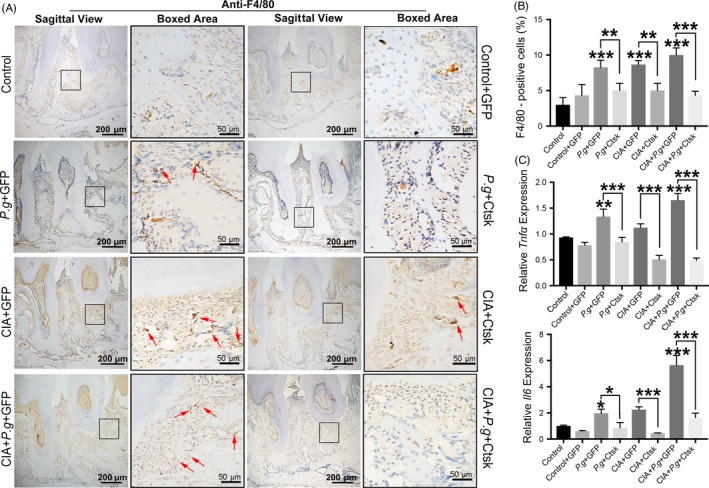
Inhibition of Ctsk in the lesion area reduced the number of macrophages and inflammatory cytokines in the periodontium. A, IHC staining of F4/80‐positive cells in periodontal tissue. The red arrow indicates positive cells. B, Quantification of F4/80‐positive cells in different groups. C, Relative mRNA expression of *Tnfα* and *Il6* in periodontal lesions detected by qRT‐PCR. Data are presented as the mean ± SD (n = 10 per group), compared with the control. Control: untreated DBA/J1 mice; *Pg*: mice orally infected with *Porphyromonas gingivalis*; CIA: mice with collagen‐induced arthritis (CIA); GFP: mice received empty AAV vectors (AAV‐U6‐CAG‐EGFP); and Ctsk: mice received AAV‐U6‐Ctsk‐shRNA‐CAG‐EGFP. *P < .05, **P < .01 and ***P < .001. The asterisk (with no line connection) on the column of this group represents the statistical difference between it and the control group. Experiments were repeated three times

We detected the infiltration of macrophages in the periodontium through IHC analysis. The specific marker of macrophages F4/80 was used as the target protein (Figure 4A). Quantitative staining results showed that in the periodontal area, the infiltration of F4/80‐positive cells was significantly increased in the periodontitis (*Pg* + GFP), arthritis (CIA + GFP) and comorbidity groups (CIA + *Pg* + GFP). The number of F4/80‐positive cells was greatest in the comorbidity group (CIA + *Pg* + GFP). When AAV‐sh‐Ctsk was applied, the number of positive F4/80 cells decreased in all groups (Figure 4B).

The results of qRT‐PCR revealed that the expression of inflammatory factors *Tnfα* and *Il6* in the periodontal area was upregulated in the periodontitis (*Pg* + GFP), arthritis (CIA + GFP) and comorbidity (CIA + *Pg* + GFP) groups (Figure 4C). When AAV was applied to inhibit Ctsk, qRT‐PCR results showed that expression of *Tnfα* and *Il6* was significantly inhibited in these groups. All these data indicated that arthritis could promote the expression of macrophages and inflammatory cytokines in periodontitis and that the inhibition of Ctsk has an anti‐inflammatory effect in the process of RA promoting periodontitis.

### 3.3 Inhibition of Ctsk in the lesion area reduced the expression of TFEB in the periodontium with RA

Previous studies have suggested that autophagy is involved in the development of periodontitis and arthritis.^15^, ^16^ In this study, inhibition of Ctsk effectively alleviated the process of promoting periodontitis by RA, which suggested that Ctsk might affect the autophagy response in the course of the disease. To evaluate this response, we first detected the classic autophagy‐coordinating protein TFEB.

**Figure 5:**
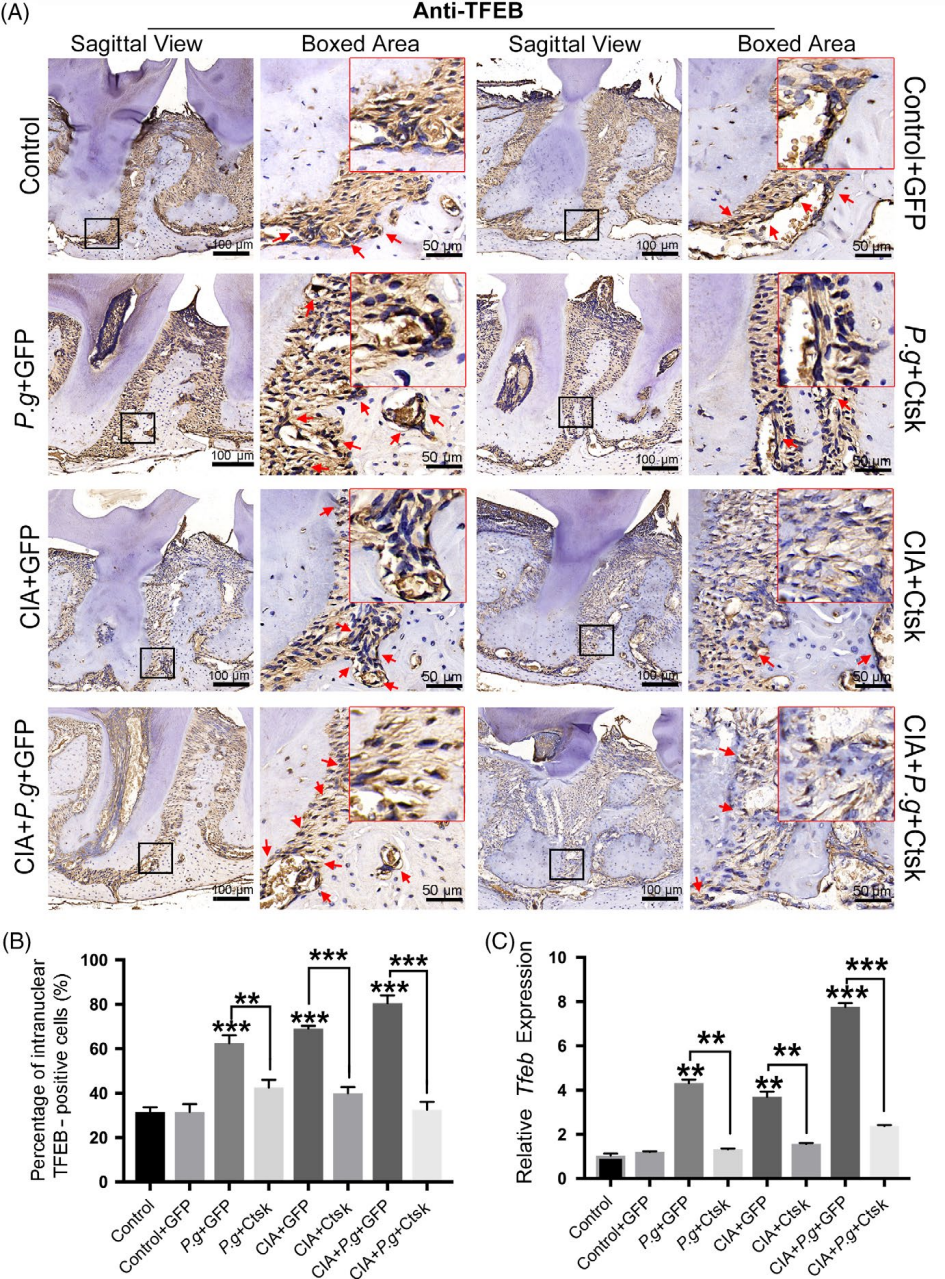
Inhibition of Ctsk in the lesion area reduced the expression of TFEB in the periodontium. A, IHC staining of TFEB‐positive cells in periodontal tissue. The red arrow indicates positive cells. B, Quantification of TFEB nuclear‐positive cells in different groups. C, Relative mRNA expression of *Tfeb* in periodontal lesions detected by qRT‐PCR. Data are presented as the mean ± SD (n = 10 per group), compared with the control. Control: untreated DBA/J1 mice; *Pg*: mice orally infected with *Porphyromonas gingivalis*; CIA: mice with collagen‐induced arthritis (CIA); GFP: mice received empty AAV vectors (AAV‐U6‐CAG‐EGFP); and Ctsk: mice received AAV‐U6‐Ctsk‐shRNA‐CAG‐EGFP. *P < .05, **P < .01, ***P < .001. The asterisk (with no line connection) on the column of this group represents the statistical difference between it and the control group. Experiments were repeated three times

The results of IHC showed that, compared with the control group, the expression of TFEB increased significantly in the periodontitis (*Pg* + GFP) and arthritis (CIA + GFP) groups, and the expression of TFEB was highest in the comorbidity group (CIA + *Pg* + GFP). As expected, the expression of TFEB was significantly downregulated after inhibition of Ctsk (Figure 5A). IHC revealed not only the expression of TFEB but also its distribution. When CTSK was inhibited, the amount of TFEB entering the nucleus decreased, and quantification of the nuclear TFEB also confirms this result (Figure 5B). The results of qRT‐PCR confirmed this finding (Figure 5C). In summary, when periodontitis was accompanied by RA, the autophagic reaction was aggravated more severely than in periodontitis or RA alone. These data also showed an association between Ctsk and autophagy, namely that inhibition of Ctsk can reduce the autophagic reaction.

### 3.4 Inhibition of Ctsk reduced the expression of the TLR9 and autophagy signalling pathway

Our previous studies also confirmed that TLR9 is highly expressed in the lesion area in periodontitis and RA. With inhibition of Ctsk, we found significantly reduced TLR9 expression in all groups.^19^ This suggests that TLR9 may be related to autophagy in the process of RA‐aggravated periodontitis, and Ctsk may also play an important role.

**Figure 6:**
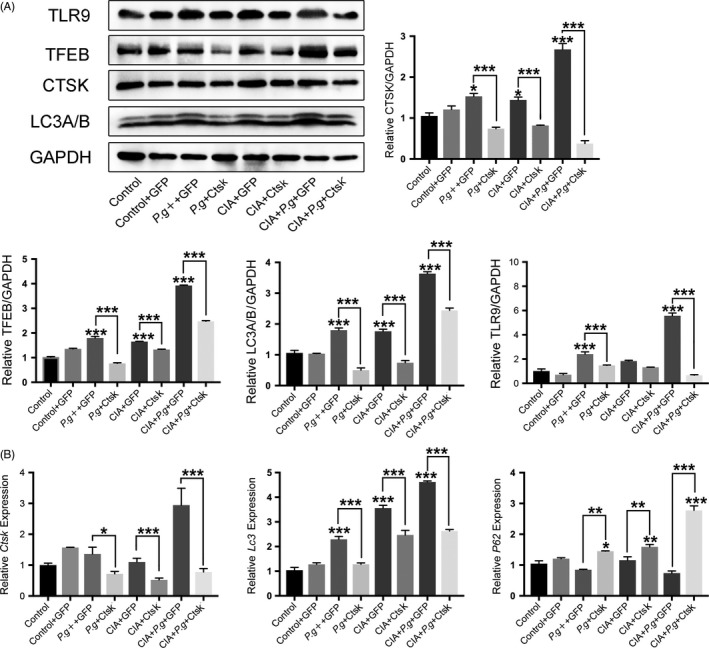
Inhibition of Ctsk reduced the expression of the TLR9 and autophagy signalling pathway. A, Western blotting analysis of TLR9, TFEB, CTSK and LC3A/B in different groups. Representative images and the normalized quantification are shown. B, Analysis by qRT‐PCR of mRNA expression of *Ctsk*, *Lc3* and *P62* in periodontal lesions. Data are presented as the mean ± SD (n = 10 per group), compared with the control. Control: untreated DBA/J1 mice; *Pg*: DBA/J1 mice orally infected with *Porphyromonas gingivalis*; CIA: mice with collagen‐induced arthritis (CIA); GFP: mice received empty AAV vectors (AAV‐U6‐CAG‐EGFP); and Ctsk: mice received AAV‐U6‐Ctsk‐shRNA‐CAG‐EGFP. *P < .05, **P < .01, ***P < .001. The asterisk (with no line connection) on the column of this group represents the statistical difference between it and the control group. Experiments were repeated three times

To test our hypothesis, we assessed expression of CTSK, TLR9 and the classic autophagy proteins by Western blotting. Expression of the proteins CTSK, TLR9, TFEB and LC3A/B in periodontal lesion areas was significantly higher in the comorbidity group (CIA + *Pg* + GFP) than in other groups (Figure 6A). The application of AAV‐sh‐Ctsk transfection not only reversed this increase in CTSK, but also decreased the expression of TLR9 and autophagy‐related proteins (Figure 6A). These results were confirmed by mRNA expression analyses (Figure 6B). As expected, RA can promote the expression of CTSK in periodontitis lesions. Expression of TLR9 and autophagy proteins also increased with increased CTSK protein, while expression of TLR9 and autophagy proteins significantly decreased with inhibition of Ctsk. These data indicated that the expression of Ctsk is connected to TLR9 and autophagy in periodontitis with RA. The trend of TLR9 expression change was the same in periodontitis with RA and autophagy, which suggested that TLR9 may be closely related to autophagy in periodontitis with RA.

### 3.5 The downregulation of autophagy induced by activation of the TLR9 pathway was mediated by Ctsk inhibition

To further investigate the roles of Ctsk and TLR9 in the immune response and autophagy, we conducted an in vitro experiment.

**Figure 7:**
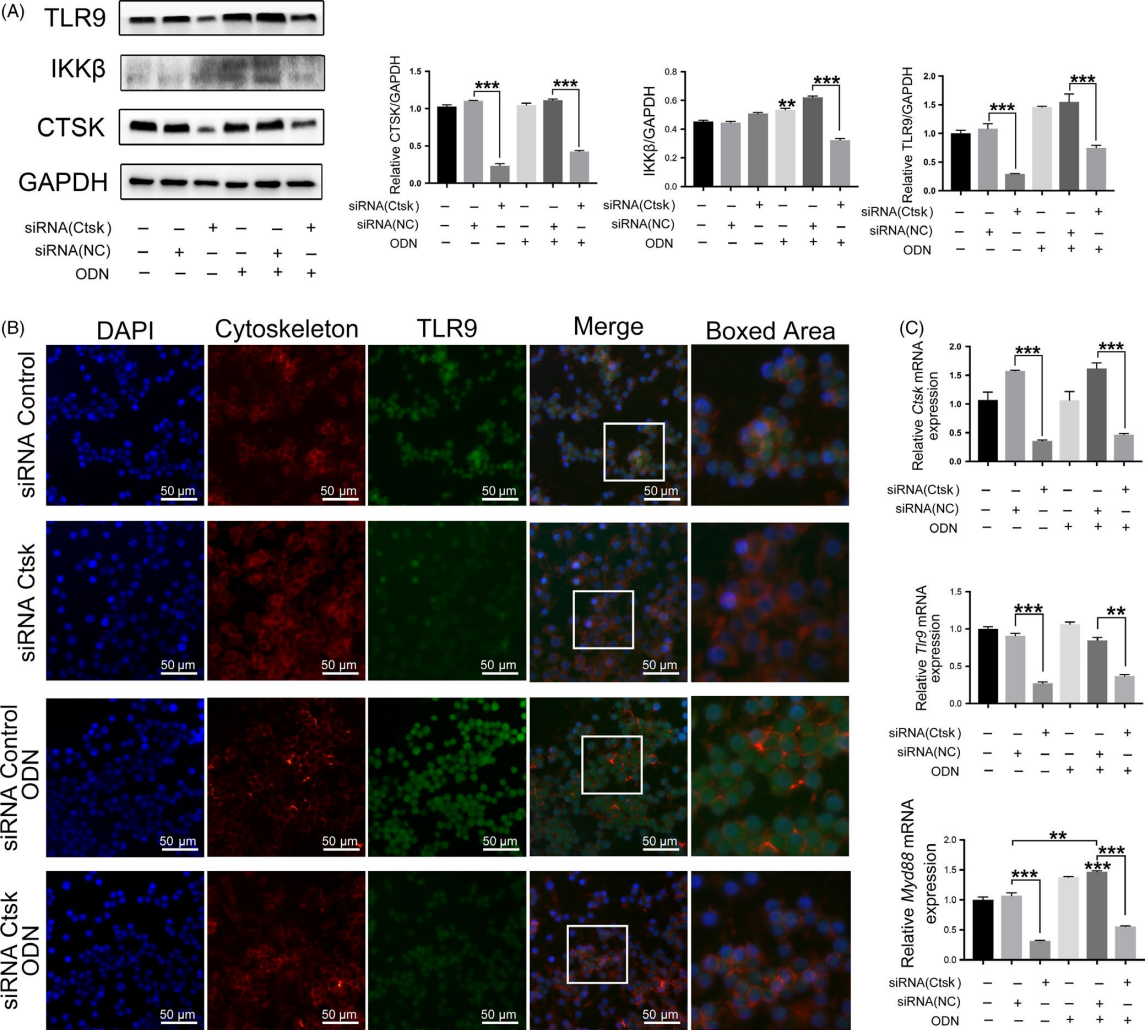
Inhibition of Ctsk reduced TLR9 signalling pathway in vitro. A, Western blotting analysis of CTSK, IKKβ and TLR9 in different groups. Representative images and normalized quantification are shown. B, IF staining of TLR9‐positive cells in different groups, and the representative images are shown. C, Analysis by qRT‐PCR of mRNA expression of *Ctsk*, *Tlr9* and *Myd88* in different groups. Data are presented as the mean ± SD, compared with the control. *P < .05, **P < .01, ***P < .001. The asterisk (with no line connection) on the column of this group represents the statistical difference between it and the control group. Experiments were repeated three times

**Figure 8:**
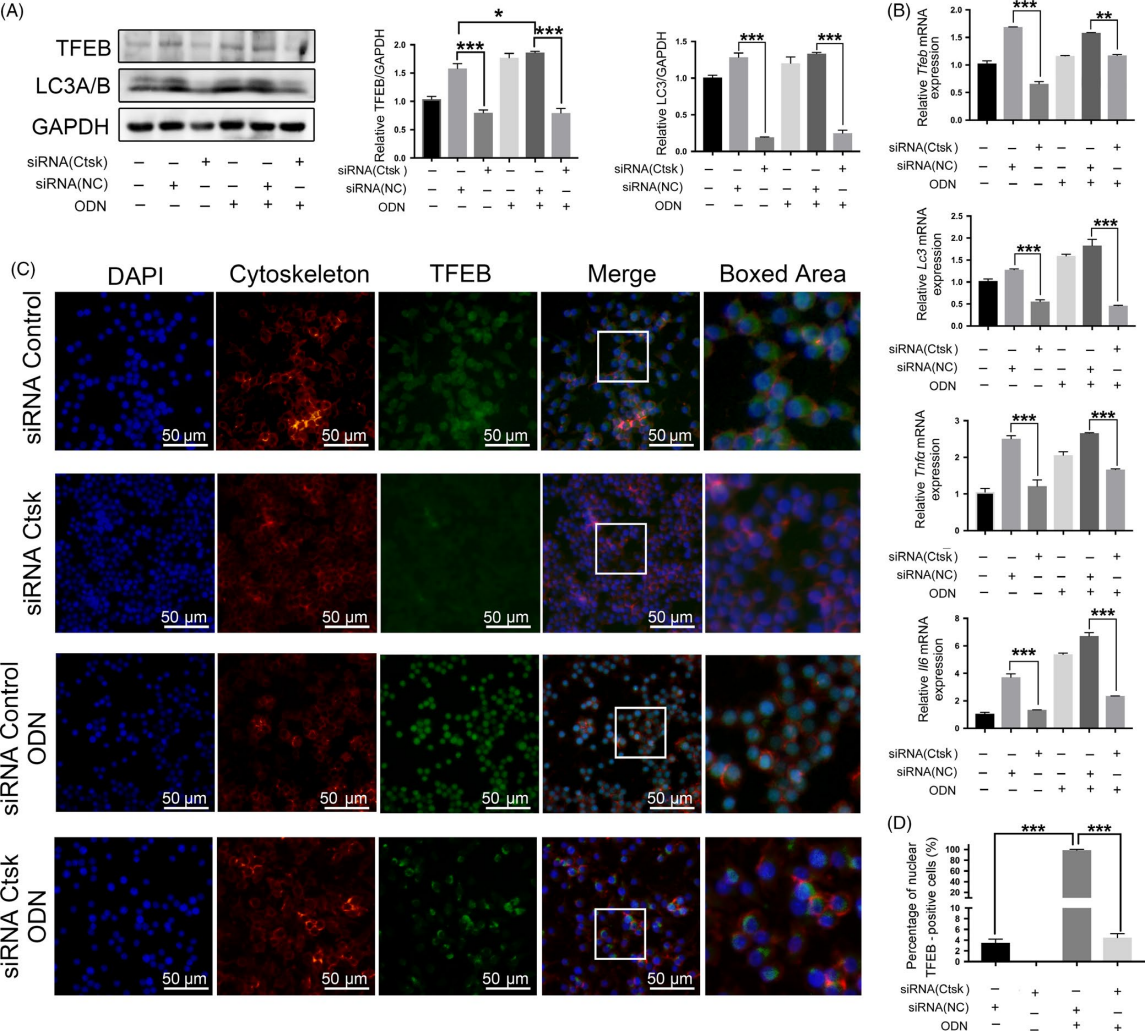
The downregulation of autophagy induced by activation of TLR9 pathway was mediated by Ctsk inhibition. A, Western blotting analysis of TFEB and LC3A/B in different groups. Representative images and normalized quantification are shown. B, Analysis by qRT‐PCR of mRNA expression of *Tfeb, Lc3*, *Tnfα* and *Il6* in different groups. C, IF staining of TFEB‐positive cells in different groups, and the representative images are shown. D, Quantification of TFEB nuclear‐positive cells in “C.” Data are presented as the mean ± SD, compared with the control. *P < .05, **P < .01, ***P < .001. The asterisk (with no line connection) on the column of this group represents the statistical difference between it and the control group. Experiments were repeated three times

Transfection of siRNA was used to inhibit Ctsk expression in macrophage cell lines. The expression of Ctsk in each group was evaluated by qRT‐PCR and Western blotting, and it was verified that siRNA is effective in inhibiting Ctsk expression at mRNA and protein levels (Figure 7A,C). Through Western blotting and IF, the expression level of TLR9 was significantly decreased with the inhibition of Ctsk (Figure 7A,B). Decreases in expression of TFEB, LC3A/B and inflammatory cytokines were also observed with inhibition of Ctsk (Figure 8A,B).

To assess the relationship between TLR9 and autophagy in vitro, CpG ODN, a specific ligand of TLR9, was employed to stimulate RAW264.7 cells. With this stimulation of TLR9, the level of TLR9 protein, its downstream protein IKKβ (Figure 7A,B) and the mRNA expression of *Myd88* (Figure 7C) were increased, despite which Ctsk did not appear to be affected by CpG ODN. According to previous studies, the TLR9‐downstream proteins IKKβ and MYD88 are related to autophagy activation.^33^, ^34^ As we predicted, the autophagy proteins, TFEB and LC3A/B, and inflammatory cytokines were increased with the activation of TLR9. However, these proteins and inflammatory cytokines still showed a downward trend with inhibition of Ctsk (Figure 8A,B).

For the key protein of autophagy, TFEB, the expression of TFEB not only varied with TLR9 and CTSK, but also its location in cells. After the activation of TLR9 by CpG ODN, IF showed that the expression of TFEB increased, and almost all of TFEB entered the nucleus to function. When the CTSK was inhibited by siRNA, the expression of TFEB decreased with the number of nucleus entering (Figure 8C), and the quantification of TFEB distribution also confirms this result (Figure 8D). It is worth noting that, when inhibiting Ctsk under the condition of TLR9 activation by CpG ODN, autophagy proteins were expressed more highly than without TLR9 activation (Figure 8).

**Figure 9:**
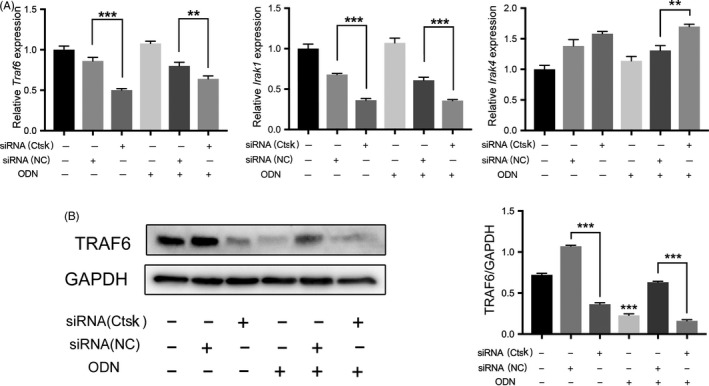
Inhibition of Ctsk reduced the expression of downstream transduction proteins of TLR9 signalling pathway. A, The relative mRNA expression of *Traf6, Irak1* and *Irak4* were detected by qRT‐PCR. B, Western blotting analysis of TRAF6 in different groups. Representative images and the normalized quantification are shown. Data are presented as the mean ± SD, repeated three times, compared with the control. *P < .05, **P < .01 and ***P < .001. The asterisk (with no line connection) on the column of this group represents the statistical difference between it and the control group

Based on these data from in vitro experiments, we infer that TLR9 is associated with autophagy in macrophage cell lines. Activation of TLR9 can activate the downstream autophagy response, while Ctsk can modulate this response. TRAF6 and IRAK1 decreased with inhibition of Ctsk (Figure 9A,B), while the mRNA expression levels of *Traf6, Irak1* and *Irak4* did not appear to be affected by TLR9 ligand (Figure 9A).

## 4 DISCUSSION

Previous studies have demonstrated a mutually promoting relationship between periodontitis and RA,^19^, ^35^ and studies on autophagy in periodontitis^36^ and arthritis are gradually accumulating.^37^ However, the role of autophagy in the mechanism underlying the link between RA and periodontitis has not been explored. Our in vivo experiment found that RA can increase the expression of autophagy proteins TFEB and LC3A/B in the periodontitis lesion area, upregulate the secretion of inflammatory factors *Tnfα* and *Il6*, promote the infiltration of macrophages and finally aggravate bone destruction. These data support the hypothesis that elevated autophagy, and its associated signalling pathways, is an important regulator of the aggravation of periodontitis by RA.

Ctsk is usually studied as a functional molecule related to osteoclasts.^38^ Our previous study showed that Ctsk could affect the TLR9 signalling pathway in periodontitis and arthritis.^19^ The present study not only verified the established connection between Ctsk and TLR9, but also indicated a novel role of Ctsk in autophagy. In periodontitis with RA, TLR9 and autophagy decreased significantly with inhibition of Ctsk. In vitro experiments showed that the autophagy response increased after TLR9 activation, while decreasing with TLR9 suppression when Ctsk was inhibited.

It has been well established that in the immune system, factors such as TLR9 recognize conserved molecular structures and initiate downstream signalling pathways to control the immune response through what is called pattern recognition reception (PRR).^39^ Early immune pathways mediated by PRR have been linked to classical inflammation, but recent studies have demonstrated a new link between PRR and autophagy.^40^ Researchers have since turn to the relationship between PRR and autophagy to explain the pathological mechanisms of immune diseases, such as periodontitis and RA. The interaction of nucleic acids with TLR9 in macrophage endocytic lysosomes is a critical step in the activation of the TLR9 signalling pathway.^41^ The existence of connections between TLRs and autophagy has been known for some time,^42^ and more recently, TLR9 was observed to play critical functions in sustaining autophagy.^43^ Myd88 and IKK complexes in the TLR9 pathway are all related to the activation of autophagy.^33^, ^34^ Most recently, studies have shown that TLR9 can regulate LC3 to directly recruit the downstream kinase IKKα.^44^ Here, we aimed to elucidate the relationship between Ctsk and downstream TLR9‐related autophagy. Our data suggest that TLR9 and autophagy proteins are upregulated in periodontitis associated with RA. Consistent with our hypothesis, transfection with AAV‐sh‐Ctsk reduced bone resorption, expression of TLR9 and autophagy status in the periodontitis and comorbid groups. In vitro, we observed an increase in autophagy after TLR9 activation, whereas inhibition of Ctsk reversed both activated TLR9 and autophagy. These results gave us reasons to explore the link among Ctsk, TLR9 and autophagy. However, blocking the TLR9 pathway and manipulating Ctsk expression could help us further observe effects of autophagy under the changes of Ctsk and come to more accurate and solid conclusions. This question will be addressed in our future studies.

We also focused on the possible role of Ctsk in the TLR9 and autophagy response in macrophages. Macrophages are important components of the innate immune system, which play the role of antigen presentation and immune regulation,^45^ and are known to play a critical role during the progress of autophagy.^46^, ^47^ Macrophages can also produce cytokines and promote the activation of T cells to secrete pro‐inflammatory cytokines, which cause tissue damage during chronic inflammation.^48^ We found that RA could aggravate the infiltration of macrophages in periodontitis lesions, while the number of macrophages was significantly decreased after inhibiting Ctsk.

In conclusion, our research provides a novel role of Ctsk‐mediated, TLR9 and autophagy in the development of periodontitis aggravated by RA. This provides a new potential therapeutic target to treat periodontitis with RA.

## CONFLICT OF INTEREST

The authors declare no potential conflicts of interest with respect to the authorship and/or publication of this article.

## AUTHOR CONTRIBUTIONS

Wei Wei contributed to conception and design; contributed to acquisition, analysis and interpretation; drafted the manuscript; critically revised the manuscript; gave final approval; and agreed to be accountable for all aspects of work ensuring integrity and accuracy. Jie Ren contributed to conception and design; contributed to acquisition, analysis, and interpretation; drafted the manuscript; critically revised the manuscript; gave final approval; and agreed to be accountable for all aspects of work ensuring integrity and accuracy. Wuwei Yin contributed to design, contributed to acquisition, critically revised the manuscript, gave final approval and agreed to be accountable for all aspects of work ensuring integrity and accuracy. Handong Ding contributed to conception, critically revised the manuscript, gave final approval and agreed to be accountable for all aspects of work ensuring integrity and accuracy. Qiuyu Lu contributed to conception, critically revised the manuscript, gave final approval and agreed to be accountable for all aspects of work ensuring integrity and accuracy. Liangyu Tan contributed to conception and design, contributed to analysis, drafted the manuscript, critically revised the manuscript, gave final approval and agreed to be accountable for all aspects of work ensuring integrity and accuracy. Shibing Deng contributed to conception, critically revised the manuscript, gave final approval and agreed to be accountable for all aspects of work ensuring integrity and accuracy. Jie Liu contributed to conception, critically revised the manuscript, gave final approval and agreed to be accountable for all aspects of work ensuring integrity and accuracy. Qin Yang contributed to conception, contributed to analysis, critically revised the manuscript, gave final approval and agreed to be accountable for all aspects of work ensuring integrity and accuracy. Jiajia Wang contributed to conception, contributed to analysis, critically revised the manuscript, gave final approval and agreed to be accountable for all aspects of work ensuring integrity and accuracy. Min Wang contributed to conception and design, contributed to analysis, critically revised the manuscript, gave final approval and agreed to be accountable for all aspects of work ensuring integrity and accuracy. Yuan Yue contributed to conception and design, contributed to analysis and interpretation, drafted the manuscript, critically revised the manuscript, gave final approval and agreed to be accountable for all aspects of work ensuring integrity and accuracy. Liang Hao contributed to conception and design, contributed to analysis and interpretation, drafted the manuscript, critically revised the manuscript, gave final approval and agreed to be accountable for all aspects of work ensuring integrity and accuracy.

## Data Availability

Research data are not shared.
